# Effect of stocking density and age on physiological performance and dynamic gut bacterial and fungal communities in Langya hens

**DOI:** 10.1186/s12934-021-01707-y

**Published:** 2021-12-04

**Authors:** Yaping Wang, Taihua Jin, Ningbo Zhang, Jiongkui Li, Yan Wang, Muhammad Fakhar-e-Alam Kulyar, Zhaoqing Han, Yongzhu Li

**Affiliations:** 1 College of Agriculture and Forestry Science, Linyi University, Linyi, 276000 China; 2 College of Veterinary Medicine, Huazhong Agricultural University, Wuhan, 430070 People’s Republic of China; 3 Qingdao Ruiyi Precision Medical Inspection Co., Ltd, Qingdao, 266000 China

**Keywords:** Stocking density, Langya laying hens, Gut microbiome, Egg quality, Age

## Abstract

**Background:**

The characterization of colonization and dynamic changes related to gut microorganisms might be vital, as it presents an opportunity to quantify the co-variation between stocking densities and gut microbiome of dynamic distribution. The objective of this study was to determine the stocking density on physiological performance and dynamic distribution of gut microbiome (including bacterial and fungal communities) of Langya laying hens in the two development stages.

**Methods:**

A randomized design with 2 × 3 factorial controls consisting of two development stages (24, 43 weeks-old) with three different stocking densities was performed. Three different stocking densities were allocated to a total of 300 11-week-old Langya laying hens (450 cm^2^/bird, 675 cm^2^/bird, 900 cm^2^/bird). Three housing densities were accomplished by raising different chickens per cage with the same floor size. The dependent variables of stocking densities at each sampling point were; growth performance, organs index, egg quality and the changes of dynamic gut bacterial and fungal communities in the cecum.

**Results:**

Results showed that the stocking density didn’t affect liver index, eggshell thickness, breaking shell strength and egg shape index. Hens from the highest stocking density had the lowest body weight, fallopian tube index, egg weight and yolk colour score. Except for the yolk colour score, the measurement changes caused by age followed the opposite pattern as stocking density. We observed a substantial rise in taxa linked with health threats when stocking density was increased, including *Talaromyces*, *Oscillospiraceae_UCG-002*, *Oscillospira*, and *Dielma*. The opposite was observed with *Bacteroides*, *Bifidobacterium*, *Lachnoclostridium*, *Eisenbergiella*, and *Kurtzmaniella*. Also, most taxa were linked to polymicrobial infection in clinical cases, especially species whose percentage declined as the hens aged, such as *Terrisporobacter*, *Faecalicoccus*, *Dialister*, *Cylindrocarpon* etc. Whereas *Sellimonas*, *Mitsuokella*, *Eurotium*, *Wardomyces* and *Cephalotheca* had the opposite trend.

**Conclusion:**

We speculated that excessive high density drove the abundance of bacteria and fungi connected with health problems. Where the gut microecology gradually reach a mature and balance status with age. Overall, this study demonstrates gut microbiome ecological processes in Langya layers at various stocking densities and finds possible connections between stocking density, microbiome and production performance. Our study will contribute to new insights associating suitable density patterns and production performance in laying hens by harnessing such a relative microbiome.

## Introduction

The poultry sector has gradually expanded its capacity to achieve greater economic returns for increasing worldwide dietary meat protein intake [1]. Consequently, stocking density has risen, which has a close link to the economic output of poultry farming. If this situation develops into overcrowding, it will have the opposite effect to the original intention, which is embodied in the following points: (1) It may synergistically limit nest, feed and water intake availability and lead to aggressive behaviour [2]. (2) It intensifies the development of feather pecking among laying hens, causing discomfort, stress and behavioural restrictions [3]. Feather pecking is birds’ lousy behaviour, initiated by aggression, leading to feather and skin damage [4]. It has also been reported that broilers' high stocking density and production performance (e.g., weight and feed efficiency) are negatively correlated [5]. This condition could be the result that high stocking density break the intestinal villi structure in broilers, causing a reduction in absorption capacity [6]. Also, the increases in stocking density cause higher intestinal permeability due to weak intestinal barrier function [7]. This is not surprising since the effect of the environment on early life is enough to persist throughout the life span [8]. The adaptive dynamic distribution of gut flora is one of the key factors attracting growing attention.

The gut microbiome, which comprises billions of microorganisms, serves as a bridge among the host's health, nutrition and survival conditions [9]. Studies have shown that the dynamic changes of intestinal microbes are the manifestation accompanying changes of various host and environmental factors [10]. It could be due to various reasons, including genetics and environmental factors, such as housing density, diet, environment and litter management etc. [11, 12]. These changes are extremely related to numerous host physiological aspects such as nutritional status and stress response [13, 14]. The manifests of gut microbiome may differ in various ecological niches of host and may vary with stocking density in laying hens. However, the foundations of microorganism-linked growth environments and the underpinnings of these relationships remain unknown. The majority of research so far has focused on the relationship between stocking density, growth performance, welfare and disease susceptibility. Whereas the impact of farming practice on gut microbiomes has been overlooked [15, 16]. Recently, various researches have demonstrated the pronounced impacts of gut microbiota on different livestock productive performances [17–19]. It conveyed a message that the intestinal microbiome has been identified as one regulator of host performance. This regulated process even included the egg production performance of laying hens, proven in previous study [20, 21]. The distinct gut communities that clone the gut of laying hens modulated the host-microbiome for production performance [20]. Adding Lactobacillus to the diet, for example, improved host-microbe interactions even resulted in increased egg production [21]. Consequently, the most abundant communities of the intestinal microbiome can be recognized as evidence of a connection among density pattern, intestinal microbiota, and production performance in laying hens. However, previous research on the microbiomes of the gastrointestinal system has focused on bacterial populations, with just a few studies taking into account the multi-kingdom characters of the integrated microbial ecology [22].

A thriving population of the intestinal community, including bacterial and fungal, lives in the host gut environment. The enormous number of bacteria co-exists with the fungi microbiomes in the gastrointestinal tract, substantially expanding the populations of the microorganisms. Increasing evidence has demonstrated the crucial relationships between the fungal population and host health [23, 24]. This relationship between fungi and bacteria has emerged as crucial to maintaining gut homeostasis [25]. Therefore, the combined characterization of fungal and bacterial populations in hens is required for investigation. Furthermore, longitudinal studies of gut content samples of different ages revealed a connection between various changes in gut microbial composition and diversity [26, 27]. For example, in animal husbandry production, young animals are susceptible to intestinal disease, e.g., chronic inflammation and diarrhea, which are highly associated with gastrointestinal dysfunction. This condition gradually improves with age [11]. As a result, the gut microbiota, which seemed to be stable, was sensitive to the host's age and health status [28]. Although evidence indicates, embryonic colonisation occurs in chicken’s intestinal microflora, colonizing naturally with microbes upon hatch [29]. Previous studies have demonstrated that early colonization of the intestinal microbiota is a dynamically evolving, non-fixed structure influenced by diet and growth environment and reaches stability after maturity [30, 31]. Therefore, there might be some unavoidable connections between changes in age and the tolerance against diseases of laying hens. However, a report on the potential law between the age-related factor and the changes in intestinal micro-ecology of the Langya laying hens is rare.

Characterizing the intestinal microbiome colonisation and succession based on density and age-dependent effects in laying hens might be necessary if microbiota-based therapeutic or disease prevention or egg-laying performance improvement strategies are adopted. Therefore, the objectives of this study were to evaluate the impacts of stocking density and age on the physiological property of Langya laying hens, focusing on growth performance, egg quality, Longitudinal and horizontal investigations of the gut microbiome. Our study will contribute to new insights associating suitable density patterns and production performance in laying hens by harnessing such a relative microbiome. We investigated the dynamic changes of density-related gut microbiomes of bacterial and fungal populations of Langya laying hens over the age at various densities by 16S ribosomal DNA (16S rDNA) and Internal Transcribed Spacer (ITS) amplicon sequencing.

## Materials and methods

### Experimental design

Commercially, 300 11-week-old Langya laying hens of similar weight, genetic background, and immune procedures were selected to participate in this experiment. All birds initially allotted to 1 of 3 stocking density treatments: (H) 450 cm^2^/bird, (I) 675 cm^2^/bird, (L) 900 cm^2^/bird were followed through further experiment analysis. During entire experiment periods, all hens had free access to water and diet. A constant room temperature of 20 °C ± 3 °C was achieved with a 16:8 light–dark cycle (h). At the beginning of the 24th (13 weeks after range access, named AL, AI and AH) and 43rd (32 weeks after range access, named BL, BI and BH), the individual bodyweight of all hens was recorded. Three birds from each group were randomly selected to be slaughtered at each sampling point. The carcass was then dissected to collect the whole liver and fallopian tubes. Then organ index relative to the hen’s body weight was calculated with following formula:

$$Organ\;Index\;\% = \frac{Weight\;of\;organ \left( g \right)}{{Weight\;of\;hen \left( g \right)}}.$$

To avoid cross-contamination of the contents of various intestinal segments, they were tied with sterile cotton thread at the intersection of the ileum and cecum. The cecum contents were quickly frozen in liquid nitrogen and then stored at − 80 °C for further high-throughput sequencing analysis.

### Egg quality detection

At each sample point, 10 eggs from each group were randomly collected for quality assessment. A total of 60 eggs (excluding unqualified and broken eggs) were collected throughout the trial. Professionals who were blinded to the treatments carried out this process by using analytical balances and quasi-static compression (Technical Services and Supplies, TSS, Dunnington, UK) to measure whole egg weight (g) and eggshell breaking strength (N). Egg yolk color was determined using TSS equipment. According to the following formula, the egg shape index was evaluated.

$$Egg\;shape\;index = \frac{{Vertical\;diameter \left( {mm} \right)}}{{Transect\;diameter \left( {mm} \right)}}.$$

The thickness of a single eggshell was calculated through the average shell thickness (mm^−2^) of the large, small and equatorial regions.

### 16S rDNA and ITS genes amplicon sequencing

According to the procedure suggested by manufacturer, the cecal microbial community DNA was extracted using QIAamp DNA Mini Kit (QIAGEN, Hilden, Germany). Bacterial universal primers, 338F (50-ACTCCTACGGGAGGCAGCAG-30) and 806R (50-GGACTACHVGGGTWTCTAAT-30) were used to amplify along with V3/V4 hypervariable regions of 16s rDNA by Polymerase Chain Reaction (PCR) [32]. The annealing temperature was 55 °C during the 30 PCR cycles. The fungal ITS gene amplification was conducted using primers ITS5F (5′-GGAAGTAAAAGTCGTAACAAGG-3′) and ITS2R (5′-GCTGCGTTCTTCATCGATGC-3′). Visualized the PCR products by 1.5% agarose gel electrophoresis and then quantified it by PicoGreen dsDNA Quantitation Reagent (Invitrogen, USA). To obtain purified PCR products, the AxyPrep DNA Purification kit was involved in this part (Axygen Biosciences, USA). The library generated by the purified PCR products were Paired end sequenced (Paired_End) based on the Illumina NovaSeq sequencing platform (Illumina NovaSeq PE250, United States). We spliced and filtered the original data to filter out contaminated data, such as chimera sequences, nucleotide mismatch, and ambiguous character reads, to obtain accurate and reliable adequate data.

### Bioinformatics and statistical analyses

The identical Operational Taxonomic Units (OTUs) were clustered and classified using Uparse software based on verified data and sequences that were ≥ 97% similarities. In addition, using the Silva and Unite databases, bacterial and fungal classifications were made. Alpha diversity was calculated using QIIME2 software using normalized OTU counts for Chao1, ACE, Shannon, and Simpson. LEfSe analysis [Line Discriminant Analysis (LDA) Effect Size] was used to distinguish significant taxa difference between different stocking density treatment groups. Beta diversity was measured by Principal coordinate analysis (PCoA) through unweight and weighted UniFrac distances. Based on the similarity of 16S rDNA and ITS gene sequences, Tax4Fun grouped OTU using the SILVA database as a reference sequence. Finally, a function annotation information was obtained. T-test was used to find all groups’ significance levels at classification levels (Phylum and Genus). The data on the organ index and egg characteristics were statistically evaluated using a T-test in SPSS Software. Probability value of < 0.05 was considered statistically significant.

## Results

### Stocking density and age impacted on growth performance and egg quality parameters of Langya hens

**Fig. 1:**
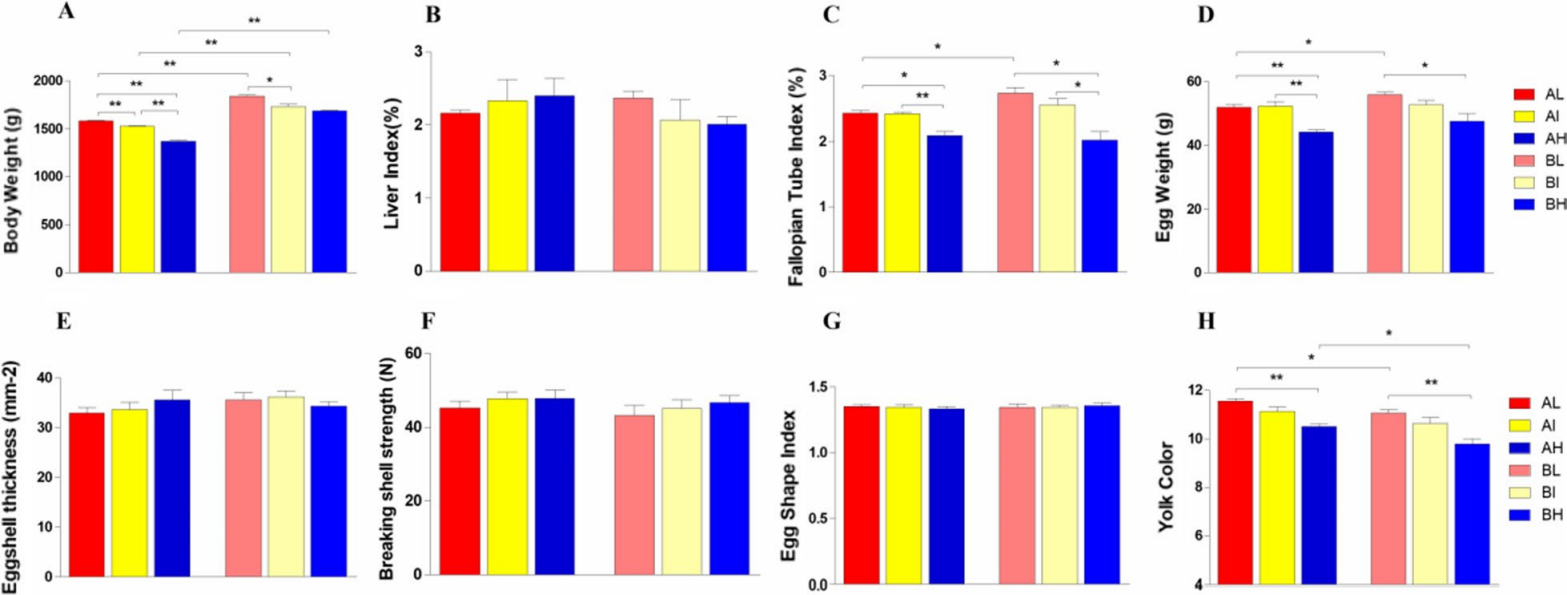
The physiological performance and egg quality measurements from Langya hens housed different stocking densities, sampled at 24th and 43rd weeks of age. AL, AI, AH represented the densities of 900 cm^2^/bird, 675 cm^2^/bird, 450 cm^2^/bird on the 24th week; BL, BI, BH represented the densities of 900 cm^2^/bird, 675 cm^2^/bird, 450 cm^2^/bird on the 43th week.*p < 0.05; **p < 0.01

Growth performance of stocking density was monitored from 11th to 43rd week. Stocking density or age did not affect any of the four indicators: liver index, eggshell thickness, breaking shell strength and egg shape index (*p* > 0.05) (Fig. 1B–G). Nonetheless, there was statistical evidence (*p* < 0.05 or *p* < 0.01) of the stocking density × day interaction for body weight, fallopian tube index, egg weight and yolk color (Fig. 1A, C, D, H). Hens stocked at the highest density tended to be the lowest fallopian tube index, body weight, egg weight and yolk color scores without regard to age. Between 24 and 43 weeks, there was a tendency for significant variations in body weight, with older hens having a greater body weight (*p* < 0.01). Except for the yolk colour score, the measurement changes caused by age followed the opposite pattern as stocking density. As a result, the impacts of stocking density and age have been clarified.

### Analysis of sequencing data and microbiome diversities

**Fig. 2:**
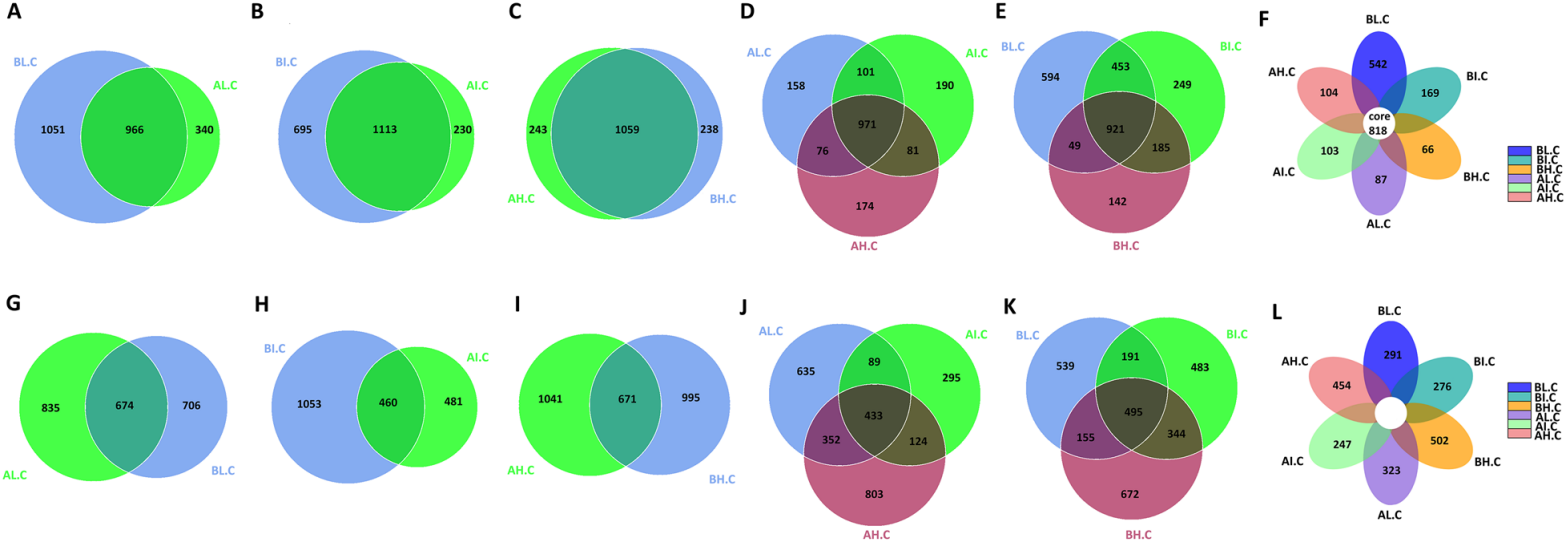
Venn diagrams analysis. The overlap in the figures represents the core OTUs among groups. **A**–**F** Represents the bacterial OTUs compositions of gut microbiome. **G**–**L** Represents the fungal OTUs compositions of gut microbiome

**Fig. 3:**
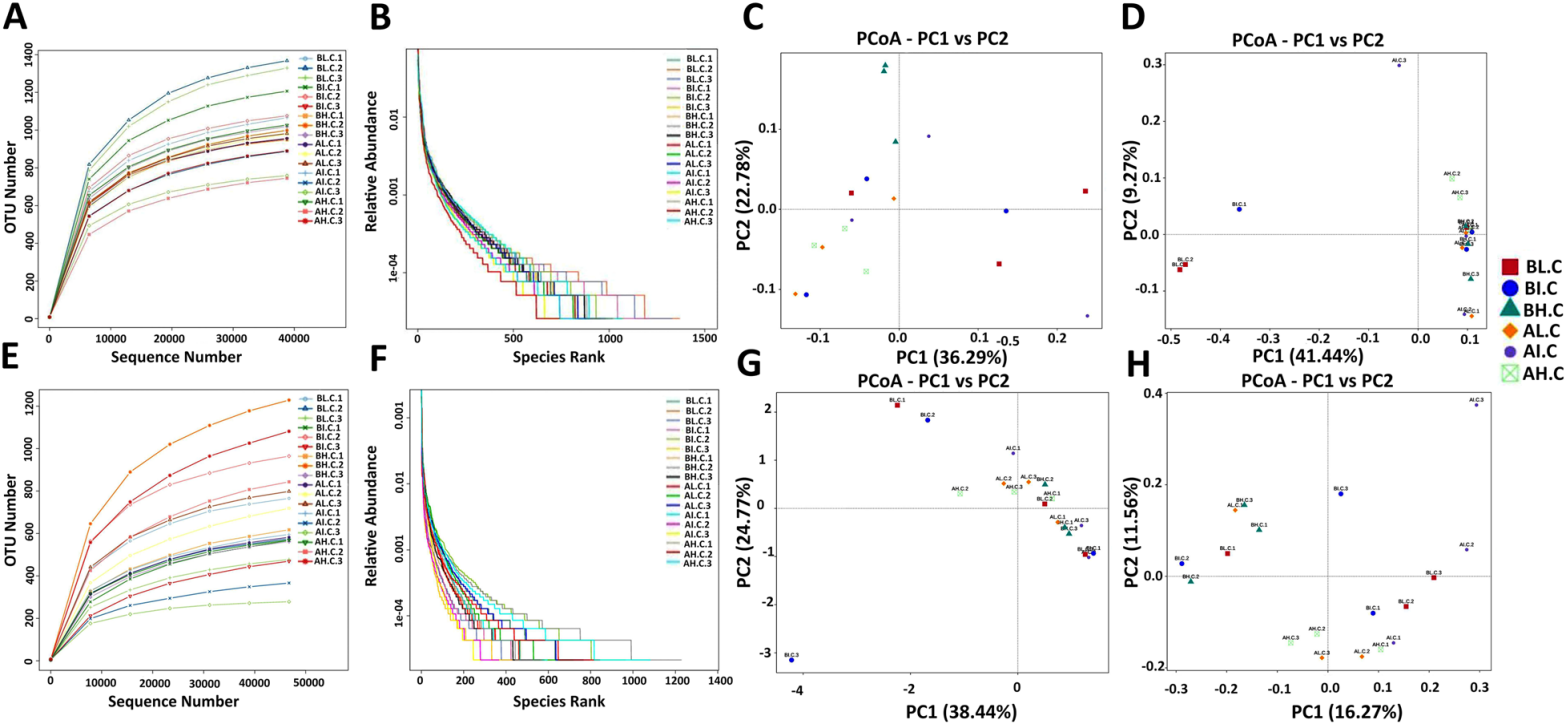
Analysis of gut microbial OTUs structures based on bacterial and fungal communities. **A**, **B** Represents the rarefaction curves and rank abundance curves of bacterial communities’ structures, respectively. **C**, **D** Represents bacterial PCoA scatterplot through weight and unweighted UniFrac distances, respectively. **E**, **F** Represents the rarefaction curves and rank abundance curves of fungal communities’ structures, respectively. **G**, **H** Represents fungal PCoA scatterplot through weight and unweighted UniFrac distances, respectively

**Fig. 4:**
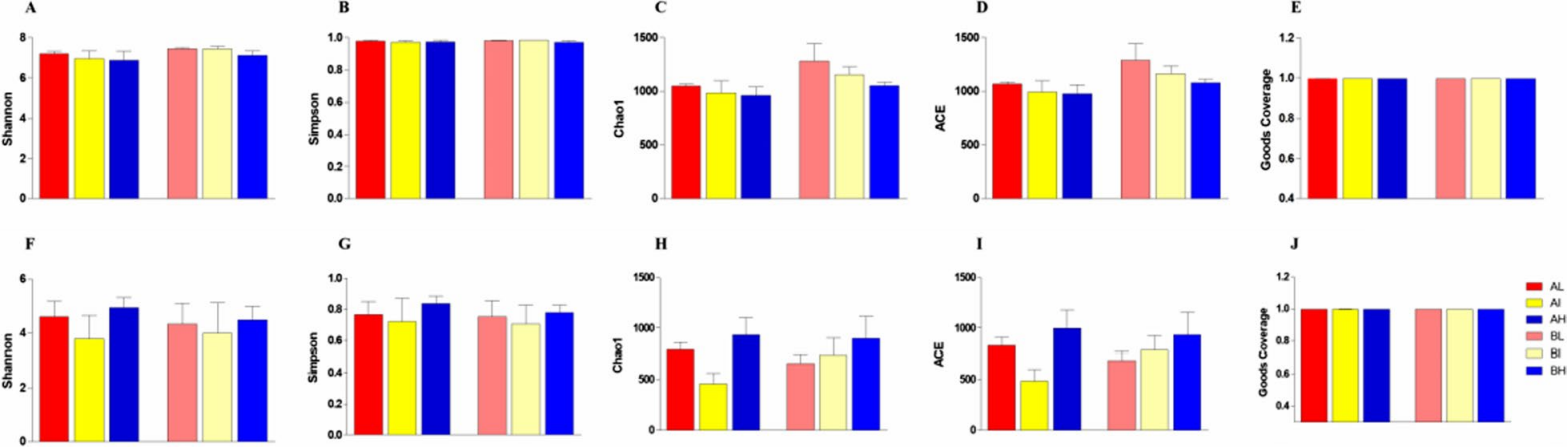
Analysis of gut bacterial and fungal diversities. **A**–**E** Represents bacterial Shannon, Simpson, Chao1, ACE, and Goods Coverage indices, respectively. **F**–**J** Represents bacterial Shannon, Simpson, Chao1, ACE, and Goods Coverage indices, respectively

The fecal samples were subjected to amplicon sequencing. A total of 1,462,162 bacterial (AL = 261,402, AI = 254,067, AH = 249,738, BL = 198,429, BI = 236,785, and BH = 261,741) and 1,439,650 fungal (AL = 247,219, AI = 252,929, AH = 255,089, BL = 206,262, BI = 257,568, and BH = 220,583) raw sequences were obtained from the V3/4 and ITS regions, respectively (Tables 1, 2). After optimizing the preliminary data, a total of 943,503 bacterial and 1,136,789 effective fungal tags were acquired from all the samples, ranging from 43,144 to 69,540 sequences per sample. In the current microbiome investigation, 9073 OTUs and 8721 OTUs, clustered at 97% sequence similarity, were recognized in the bacterial and fungal communities, respectively (Fig. 2A–C, G–I). Moreover, 818 and 246 core OTUs were detected from the bacterial and fungal communities, which accounted for approximately 9.02% and 2.82% of the total OTUs (Fig. 2F, L). Remarkably, the number of core OTUs in total samples was much smaller than the number of unique core OTUs in any age stage. It suggests that the gut microbiome is diversified driven by age (Fig. 2D–F, J–L). Both rarefaction and rank abundance curves tended to be stable, suggesting that almost all the fungal and bacterial species were identified in the cecal contents of Langya laying hens (Fig. 3A, B, E, F). Additionally, Good’s coverage estimates were over 99% per sample, suggesting that the evenness and abundance of all samples were satisfactory (Fig. 4E, J).

**Table 1:** Sequence data of all samples

Sample name	Raw tags	Clean tags	Effective tags	Effective%
AL.C.1	81,501	76,713	50,931	61.15
AL.C.2	90,638	86,159	56,983	62.00
AL.C.3	89,263	84,533	55,539	61.28
AI.C.1	86,406	82,346	56,656	64.62
AI.C.2	78,112	73,652	53,178	65.46
AI.C.3	89,549	85,742	57,289	61.77
AH.C.1	91,920	87,595	58,169	62.37
AH.C.2	81,562	78,189	51,740	62.65
AH.C.3	76,256	73,831	50,073	64.77
BL.C.1	66,161	64,627	43,144	64.28
BL.C.2	67,401	64,723	47,031	61.66
BL.C.3	64,867	62,171	43,897	60.02
BI.C.1	65,609	62,930	46,158	61.90
BI.C.2	88,597	84,149	52,574	58.26
BI.C.3	82,579	76,377	50,811	60.56
BH.C.1	79,822	76,193	50,480	62.24
BH.C.2	87,230	84,596	59,040	66.83
BH.C.3	94,689	90,637	59,810	62.23

**Table 2:** Fungal sequence data of all samples

Sample name	Raw tags	Clean tags	Effective tags	Effective%
AL.C.1	86,266	81,169	61,170	64.68
AL.C.2	77,169	71,596	69,540	79.71
AL.C.3	83,784	79,748	60,048	65.53
AI.C.1	82,094	75,557	67,248	71.87
AI.C.2	95,455	93,870	64,786	66.15
AI.C.3	75,380	74,653	68,840	85.93
AH.C.1	100,967	96,138	68,358	63.03
AH.C.2	79,102	67,937	66,282	74.44
AH.C.3	75,020	69,746	65,565	77.26
BL.C.1	76,607	74,973	68,924	84.92
BL.C.2	65,309	58,556	47,446	61.95
BL.C.3	64,346	63,602	60,482	88.01
BI.C.1	93,075	88,406	65,185	66
BI.C.2	55,226	50,149	49,079	72.55
BI.C.3	109,267	106,696	67,547	59.7
BH.C.1	79,176	75,776	64,256	64.29
BH.C.2	64,280	63,956	61,734	61.24
BH.C.3	77,127	72,934	60,299	70.4

The effective sequences were aligned to dissect alpha and beta diversities to understand further alternations of bacterial and fungal communities in the cecum, driven by density regime and age. Analysis of alpha diversities intuitively showed no significant alteration in bacterial and fungal diversities with housing pattern and age. We speculated that the gut microbiota probably evolves into a steady ecological niche at an earlier age in laying hens. To evaluate beta diversity, we used PCoA, which reflect variabilities and similarity in samples in the taxonomic structure of the microbiome. Both the weighted and unweighted scatterplot from PCoA showed that the compositions of the gut microorganisms were relatively divergent at lower densities housing patterns and began to cluster together over the higher stocking density (Fig. 3C, D, G, H).

### Significant alterations in gut microbiome structures at different stocking densities

**Fig. 5:**
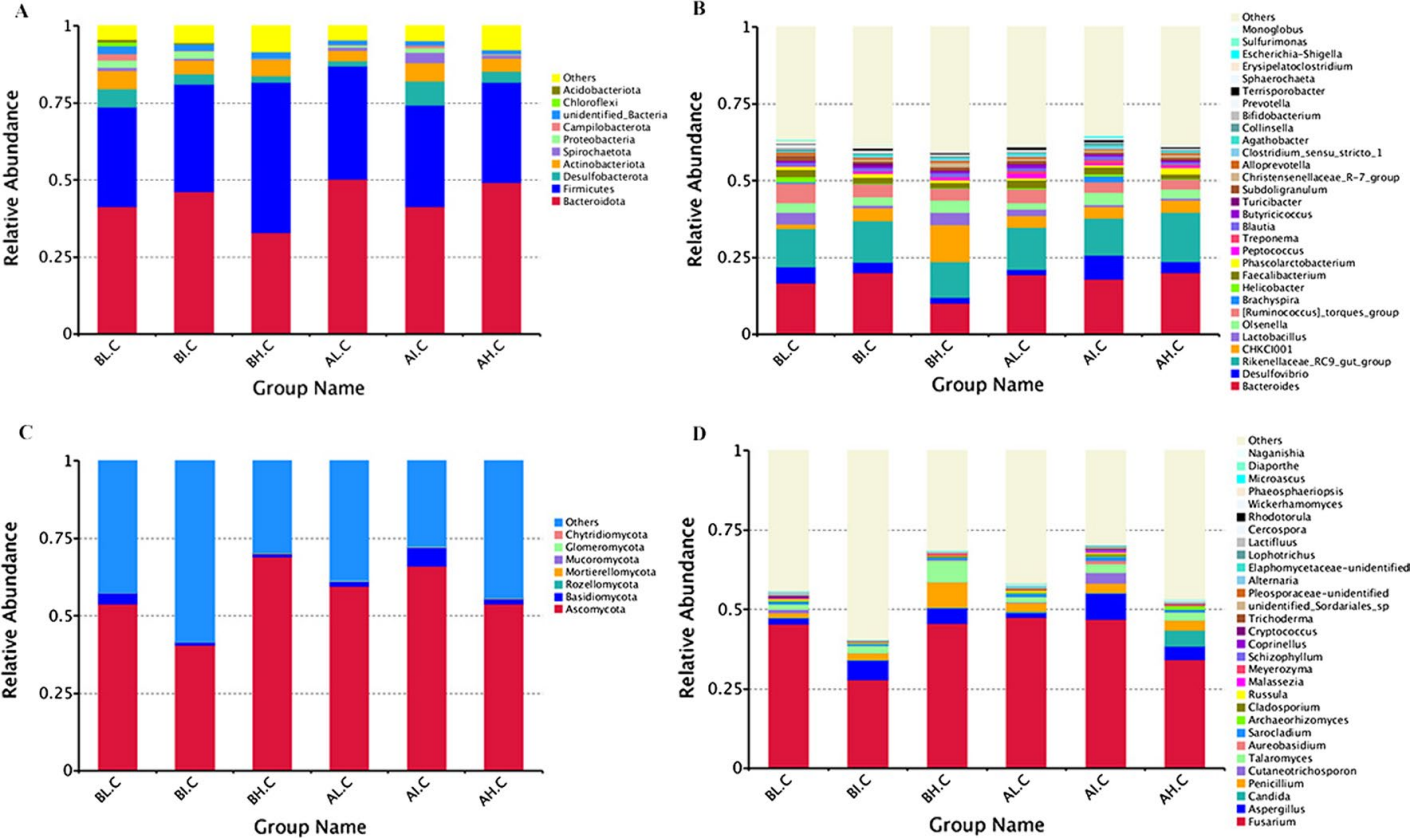
Analysis of gut microbial composition and relative abundance based on bacterial and fungal communities. **A**, **B** Represents the gut bacterial composition and relative abundance at the phylum and genus levels, respectively. **C**, **D** Represents the gut fungal composition and relative abundance at the phylum and genus levels, respectively

We analyzed the relative alterations of preponderant taxa at phylum and genus taxonomical levels in the gut’s bacterial and fungal communities. After the bacterial communities were taxonomically assigned, Firmicutes and Bacteroidetes were the dominant phyla regardless of stocking densities treatment or age, which consisted of over 36% of total tags on average (Fig. 5A). Bacteroidetes was the predominant phylum of total sequences, consisting of over 43.67% of total tags, whereas Firmicutes was secondary (36.4%). Other phyla such as Desulfobacterota, Actinobacteriota and Spirochaetota were identified in low abundance in AL (1.6%, 3.5%, 1.0%), AI (7.8%, 6.0%, 3.3%), AH (3.5%, 4.2%, 1.0%), BL (6.0%, 6.0%, 1.0%), BI (3.4%, 4.6%, 0.6%), and BH (2.0%, 5.3%, 0.3%) groups, respectively. Following the genus taxonomical level classified results (Fig. 5B), *Bacteroides* and *Rikenellaceae RC9 gut group*, and *Ruminococcus torques group* were the most preponderant genera among all groups (AL: 19.5%, 13.8%, 4.4%; AI: 18.0%, 12.1%, 4.1%; AH: 20.2%, 16.0%, 3.3%; BL: 16.7%, 12.4%, 6.3%; BI: 20.1%, 13.5%, 4.3%; BH: 10.1%, 11.6%, 4.1%). Besides these three dominant genera, the abundantly present genera were *Desulfovibrio* (5.2%) and *Lactobacillus* (3.8%) in the BL group, while *Desulfovibrio* (3.3%) and *Olsenella* (3.9%) were observed as the predominant genera in the BI and BH groups, respectively. Additionally, the top 10 phyla and top 30 genera of cecal fungal community in all groups were presented in Fig. 5C, D. According to the results of taxonomic classification of gut fungal OTU preponderant taxa, there were total 7 phyla (Ascomycota, Basidiomycota, Rozellomycota, Mortierellomycota, Mucoromycota, Glomeromycota, and Chytridiomycota) identified in the fungal communities of cecal contents. Ascomycota, which accounted for more than 40% of total tags on average in all samples, was the most dominant phylum regardless of age and stocking density. At the genus level, the most prevalent genera were *Fusarium* and *Aspergillus*, which were found in AL (47.6%, 1.4%), AI (46.8%, 8.2%), AH (34.2%, 4.2%), BL (45.4%, 2.0%), BI (28.0%, 6.0%), and BH (45.6%, 4.9%) groups, respectively.

### Significant alterations in gut communities’ composition and variability at different stocking densities

**Fig. 6:**
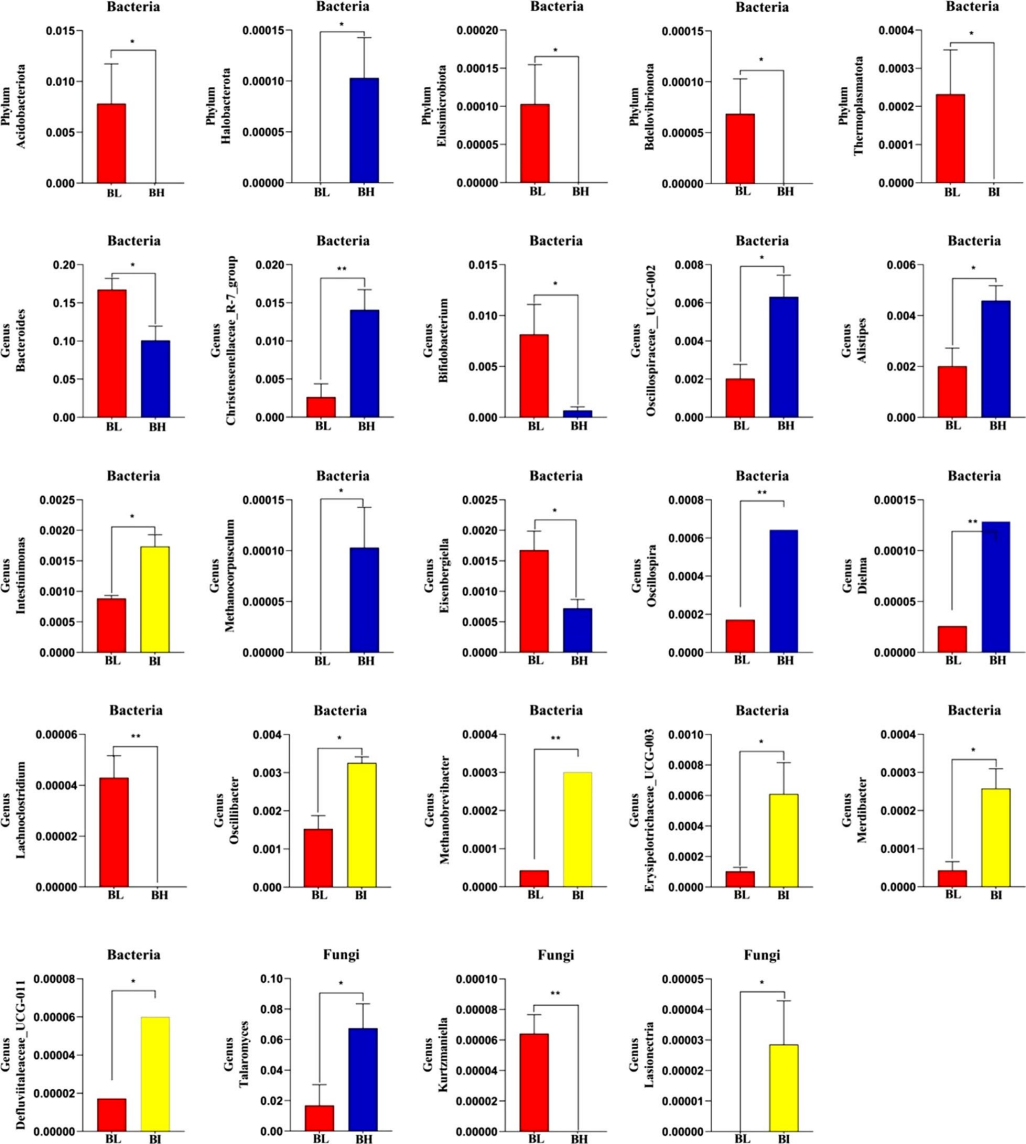
Significant alterations in gut community composition and variability based on bacterial and fungal communities at different stocking densities. *p < 0.05; **p < 0.01

We used Metastats to assess the differentially abundant classifications across groups to learn more about how the bacterial and fungal taxonomic compositions at the phylum and genus levels changed as the stocking densities. When the relative abundance of microorganisms at the phylum level were plotted on a histogram, certain taxonomic differences were found to be clustered through housing density (Fig. 6). When assessing an exploratory comparison of phyla taxa between BL and BH, four phyla relative abundances had significantly altered phenotypes. Acidobacteriota, Elusimicrobiota, and Bdellovibrionota were enriched in BL, while Halobacterota was enriched in BH (all: *p* < 0.05). Additionally, phylum Thermoplasmatota even cannot be found in the cecal contents of BI group. *Bacteroides* (*p* < 0.05), *Bifidobacterium* (*p* < 0.05), *Eisenbergiella* (*p* < 0.05), and *Lachnoclostridium* (*p* < 0.01) were significantly higher in BL relative to BH, whereas the opposite was observed with *Christensenellaceae_R-7_group* (*p* < 0.01), *Oscillospiraceae_UCG-002* (*p* < 0.05), *Alistipes* (*p* < 0.05), *Methanocorpusculum* (*p* < 0.05), *Oscillospira* (*p* < 0.01), and *Dielma* (*p* < 0.01). At the genus level, the relative abundances of *Intestinimonas* (*p* < 0.05), *Oscillospira* (*p* < 0.05), *Oscillibacter* (*p* < 0.05), *Methanobrevibacter* (*p* < 0.01), *Erysipelotrichaceae_UCG-003* (*p* < 0.05), *Merdibacter* (*p* < 0.05), and *Defluviitaleaceae_UCG-011* (*p* < 0.05) were significantly higher in BI and lower in BL.

Notably, the fungal communities revealed a pattern that differed from that of gut bacterial communities. The results of fungal communities at the phylum level showed no significant change in the compositions and proportions of phyla at different housing densities of Langya laying hens, further suggesting the relatively stable gut fungal communities in hens (Fig. 5C). Fungal genera with substantial housing density-related alternations were identified using genus-level cluster analysis. Results showed that at the genus level, the abundance of *Talaromyces* (*p* < 0.05) in the BH was significantly higher than in the BL group. In contrast, the *Kurtzmaniella* content was lower (*p* < 0.01). The relative abundance of *Lasionectria* was significantly enriched in the BI than BL (*p* < 0.05).

### Significant alterations in gut communities’ composition and variability with age

**Fig. 7:**
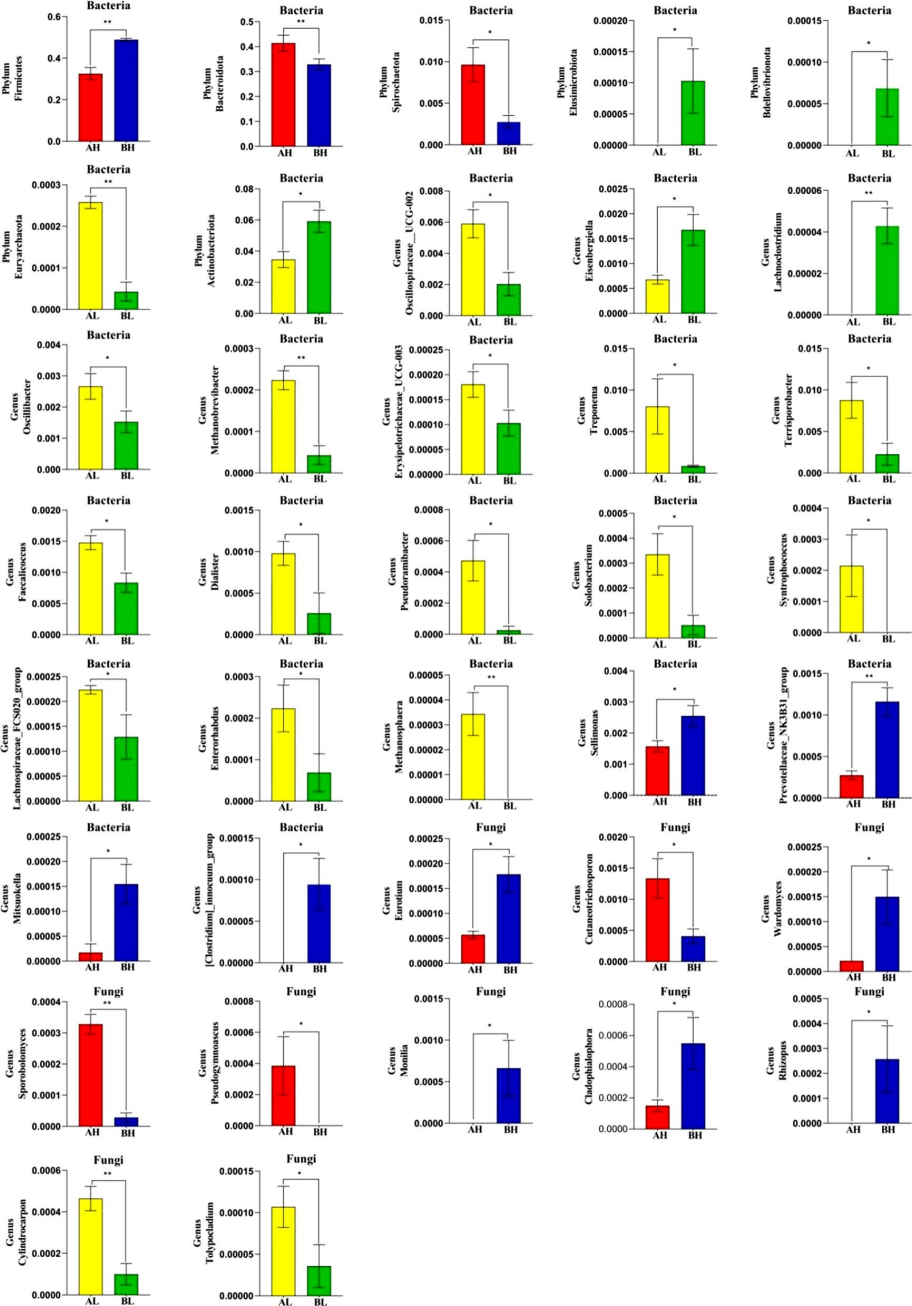
Significant alterations in gut community composition and variability based on bacterial and fungal communities with age. *p < 0.05; **p < 0.01

We then evaluated how gut bacteria and fungi communities vary overage using Metastats analysis (Fig. 7). Following the results of phylum assignment, both Bacteroidota (*p* < 0.01) and Spirochaetota (*p* < 0.05) were demonstrated to have higher abundances in AH and Firmicutes (*p* < 0.01) was higher in BH. Elusimicrobiota, Bdellovibrionota, and Actinobacteriota were lower in AL (all: *p* < 0.05), and Euryarchaeota was lower in BL (*p* < 0.01). At the genus level, the major changes included decrease in the *Oscillospiraceae_UCG-002* (*p* < 0.05), *Treponema* (*p* < 0.05), *Terrisporobacter* (*p* < 0.05), *Faecalicoccus* (*p* < 0.05), *Dialister* (*p* < 0.05), *Pseudoramibacter* (*p* < 0.05), *Solobacterium* (*p* < 0.05), *Syntrophococcus* (*p* < 0.05), *Lachnospiraceae_FCS020_group* (*p* < 0.05), *Enterorhabdus* (*p* < 0.05), *Methanosphaera* (*p* < 0.01) of cecum contents as the hens aged (AL vs. BL). In contrast, some specific taxa were significantly increased with age. For example, *Sellimonas* (*p* < 0.05), *Prevotellaceae_NK3B31_group* (*p* < 0.01), *Mitsuokella* (*p* < 0.05), and *[Clostridium]_innocuum_group* (*p* < 0.05) were higher in BH and lower in AH. Notley, several classifications varied simultaneously according to stocking density and age. The abundance of the *Eisenbergiella* and *Lachnoclostridium* remained high in the lowest density, decreased in the BH (BL vs. BH, *p* < 0.05 and *p* < 0.01), and increased in the cecum as the laying hens aged (AL vs. BL, *p* < 0.05 and *p* < 0.01). *Oscillibacter*, *Methanobrevibacter*, and *Erysipelotrichaceae_UCG-003* were abundant in the cecum of BI, decreased in the BL (BL vs. BI, *p* < 0.05 or *p* < 0.01), and subsequently decreased with age (AL vs. BL, *p* < 0.05 or *p* < 0.01). Several significant differences at the genus level were noted in the relative abundance of the gut fungal microbiome at different age periods. A comparison of the AL and BL showed significant enrichment in the abundance of *Cylindrocarpon* (*p* < 0.01) and *Tolypocladium* in the former (*p* < 0.05). Additionally, compared with BH, the relative abundances of *Cutaneotrichosporon* (*p* < 0.05), *Sporobolomyces* (*p* < 0.01), and *Pseudogymnoascus* (*p* < 0.05) increased significantly in AH, while *Eurotium* (*p* < 0.05), *Wardomyces* (*p* < 0.05), *Monilia* (*p* < 0.05), *Cladophialophora* (*p* < 0.05), *Rhizopus* (*p* < 0.05), and *Cephalotheca* (*p* < 0.01) were opposite.

## Discussion

According to previous research, different stocking densities were achieved in this experimental procedure by housing a different number of hens in cages with the same floor space [7]. The interactions between gut microorganisms and the host’s trillions have been considered significant modulators in health or disease states. Many factors, including diet, growth environment conditions, and physical activity, can negatively impact the dynamically evolving gut microbiota [30, 31]. Several studies have shown that the micro-ecology of the intestine develops with age and achieves stability beyond maturity, which develops into a restricted ecological niche [33, 34]. Hence, there might be some unavoidable correlations among the stocking density, age factor and gut microbiota change. Still, the particular connections are unclear. Most previous studies evaluated the effects of stocking density on hens feed intake, mortality, body weight, and production [35, 36]. The current research is just a few that evaluates the effects of stocking density and age factor on growth performance, organ indexes, egg quality, and dynamic distribution of gut microbiome, including bacterial and fungal communities of Langya laying hens. Our results indicated that stocking density didn’t affect liver index, eggshell thickness, breaking shell strength, and egg shape index. Hens from the highest stocking density had the lowest body weight, fallopian tube index, egg weight, and yolk colour score. Except for the yolk colour score, the measurements changes driven by age had the opposite trend to stocking density. Moreover, the excessive increase in the stocking density of laying hens exacerbates adverse effects on the gut microbiome. There was a substantial difference in dynamic changes in the microflora composition and structure with changed stocking density and age. That might be linked to physicochemical conditions and substrate usability in the intestine under different situations.

Stocking density has always been identified as a major factor affecting hens’ performance. Previous studies suggested that the production performance, egg quality, liver lipid reserves, and intestinal barrier function are normally influenced by density patterns, which revealed a nonlinear relationship [7, 37]. It’s no doubt that the bodyweight significantly increased with age in this study. Notably, increasing housing density significantly decreased body weight, fallopian tube index, and egg weight but had no adverse effects on the liver index (stocking density: the highest vs. the lowest). However, there were no significant changes in egg weight, fallopian tube, and liver indexes between the lowest and middle stocking densities, indicating that the middle stocking density might have no detrimental effects on the production performance of laying hens. However, it was likely that the highest stocking density of 450 cm^2^/bird decreased the growth performance of laying hens. The current result disagreed with previous analysis reporting that the stocking density of over 25.3 birds/m^2^ had no negative effects on broiler growth performance [7]. The inconsistence in the upper limits of housing density among those studies might result from the alterations in the experimental scheme, such as gradations of stocking densities and hen species [38]. Previous analysis suggested that laying hens with higher stocking density spent less time on feed intaking [39]. It was demonstrated that increasing housing density decreased space availability, which might negatively affect body weight gain, egg weight, etc., in layers, consistent with current observations [40]. In view of the above, we hypothesized a connection between density pattern and hens’ growth performance that might explain why hens with high stocking density had less body weight.

The normal development and functioning of the oviducts have indispensable effects on the hen’s laying abilities, which are also crucial for the egg white proteins synthesis and secretion [41, 42]. As previously mentioned, environmental growth stress modified the procedures of egg white proteins expression [42]. Even any changes in the maturity of the oviducts can directly affect egg quality [43]. This information conveyed that the excessive increased stocking density of laying hens exacerbates the negative effect on egg quality. In addition, we found that high stocking density and age didn’t significantly affect the eggshell thickness, breaking shell strength, and egg shape index, which was inconsistent with the previous report [44]. These inconsistencies might be attributed to the different growth environments or varieties of laying hens. The yolk colour was mainly dependent on xanthophylls [45]. A darker yolk in the lowest stocking density system was observed. It has not previously been explored the effects of housing density on the dry matter content of yolk [46]. Therefore, the factors that significantly affect fluctuations in egg yolk colour require further investigation.

The effect of individual species of microorganisms in certain diseases has been known, while the combined impact of the complex microbiome that connects host-gut communities interactions to health-related results have been stressed only in the past decade [47]. For example, the intestinal microbiome has been associated with numerous conditions, some of which are truly surprising [48, 49], whereas several are predictable (inflammatory bowel disease and irritable bowel syndrome) [50, 51]. Goo et al. [7] reported that high housing density could increase intestinal permeability, while dysfunction in the intestinal barrier has been identified as the probable reason for decreased growth performance and growing incidences of the disease condition. The results of growth performance in the current study supported the previous conclusion. Many stressful situations damage the intestinal barrier, including environmental stress, heat stress, and strenuous exercise [52]. The excessive-high stocking density is likely one of the factors related to elevated stress of laying hens. The above statistics show that stocking density has a significant impact on the development of the host's intestine, even mapping it onto the dynamic distribution of the gut microbiome. This study showed a substantial difference in dynamic changes in the microflora composition and structure with changed stocking density and age. That might be linked to physicochemical conditions and substrate usability in the intestine under different situations. The samples from diverse stocking densities and ages allowed us to understand how hens’ gut microbiomes fluctuate and react to different stocking levels within a developmental stage. Jami et al. [53] indicated that the diversity of gastrointestinal increased toward a mature microbial ecological niche with age. Similarly, Hu et al. [34] reported that the gut microbiome evolved into a more restricted niche as the musk deer aged. Unexpectedly, the current study observed that the differences of microbial alpha diversity were not significant regardless of age or stocking density, which was inconsistent with previous reports. As a result, we hypothesized differences in the diversity of the gut microbiota among different species. In contrast, the gut bacterial and fungal communities of Langya laying hens may evolve into a steady niche at an earlier age. Although there were no significant shifts in alpha diversity across various stocking density groups with age, the structure and composition of cecum microbiota changed.

Although the gut microbiome is essential to maintain the health state, increasing evidence suggests it probably also induces the pathogenesis of various diseases, e.g., diabetes mellitus and colon cancer [54]. Abundant beneficial microorganisms living in the gut environment are actively involved in regulating intestine functions, reducing incidences of gut diseases and driving the evolution of the immune system [55, 56]. This evidence conveyed the message that the gut environment is more sensitive to diseases, which causes a decrease in beneficial microflora or decreased potential beneficial microorganisms that exacerbate intestinal disease susceptibility. Our data showed a continuous decline in the abundances of phyla Acidobacteriota, Elusimicrobiota, Thermoplasmatota, and Bdellovibrionota with increased stocking density, whereas phyla Halobacterota had the opposite trend. Bdellovibrionota, well known as gram-negative bacterial predators, existed in various environments. The first Bdellovibrio was found in 1965, with two processes in the predation process: capitulation and degradation of the gram-negative bacterial cell wall and digesting cellular components [57]. Superoxide dismutases (SODs) were essential regulatory enzymes for microorganisms to degrade superoxide, whose one of the three separate protein genes encodes for SOD enzymes (detected in Thermoplasmatota and Actinobacteriota) [58]. Despite phylum, Acidobacteria was widespread on the planet; we still had little rudimentary information on those functions. It appeared to be helpful in carbohydrate utilization [59]. In the current study, 4 bacterial phyla (Acidobacteriota, Elusimicrobiota, Bdellovibrionota, and Thermoplasmatota) and 1 fungal species (*Kurtzmaniella*) even cannot be detected in the cecum microbiome of hens in the highest stocking density, which suggests that these microorganisms might not adapt to the gut environment. These results suggested a particular microbial community for a certain intestinal environment co-existing symbiotically with the host [60]. Further evaluation of the gut microbiome alterations is needed to thoroughly investigate the role of factors such as stocking density and age in laying hen microbiota acquisition.

Distinct density effects were also observed in the gut communities at the genus level. As increased stocking density (BL vs. BH), we observed a significant increase in detrimental taxa, including *Oscillospiraceae_UCG-002*, *Oscillospira*, and *Dielma*, whereas the opposite was observed with *Bacteroides*, *Bifidobacterium*, *Lachnoclostridium*, and *Eisenbergiella*. An important anaerobic genus, *Bacteroides*, are responsible for breaking down polysaccharides in the gut ecosystem [61]. *Oscillospiraceae_UCG-002*, *Oscillospira*, and *Dielma* significantly enriched the gut of hens in the highest density, which might be an unfavourable signal because they pose a threat to host health [62–64]. Because of the alteration of the microbiome, the in vivo findings revealed that the oral administration of *Bifidobacterium* might induce cancer immunotherapy [65]. Similarly, the relative abundance of *Lachnoclostridium* and *Eisenbergiella* enriches the hens gut at the lowest stocking density, which is linked to higher gestational weight gain [66]. Remarkably, the percentage of *Christensenellaceae_R-7_group*, *Alistipes*, and *Methanocorpusculum* in hens in the highest density was significantly increased compared with the lowest density. *Alistipes*, a member of the family Rikenellaceae, has been thought to degrade plant-derived polysaccharides [67]. *Christensenellaceae_R-7_group* and *Methanogens* aid in releasing energy intake by adipose tissue, fulfilling the energy demands of the host [68]. These results indicated that the dynamically intestinal microbes were gradually adapting to the specific intestinal environment to fulfil the needs of normal physiological function and regulate host health. Most noteworthy, altered abundances of several bacterial species contributed to signify the unique gut microbiome in BL, the prominent being the significant enrichment of the *Oscillibacter*, *Merdibacter*, *Methanobrevibacter*, and *Defluviitaleaceae_UCG-011*. *Merdibacter* genus exhibited a potential role in harvesting energy and nutrients in the gut environment, consistent with the function in metabolic homeostasis in the human gut [69]. The *Methanobrevibacter* genus, which contained the most abundant methanogenic archaea in animals, increased energy absorption efficiency by cooperating with hydrogen-producing Hydrogenoanaero microorganisms highly enriched in the obese human gut [70]. *Defluviitaleaceae UCG-011* was reported to play a major part in response to alterations in intestinal luminal proteins [71]. At the same time, *Oscillibacter* was involved in the utilization of carbohydrates and proved to increase rumen fermentation in calves [72]. Although there was an increase in relative abundances of microbial species associated with threats to host health in hens at the highest stocking density, the medium density seems to have no adverse effect on the development of gut microbes. These results consisted of the evaluation of growth performance in the current experiment. However, limited information about the impact of stocking density on the fungal communities in hens, dynamic changes of the abundance and proportion have been detected, with a minor contribution. Our data showed that high density caused an increase in the abundance of *Talaromyces* in the BH and a decrease in the abundance of *Kurtzmaniella*. Li et al. [24] reported that the abundance of *Talaromyces* was significantly higher in the diarrhea yak compared with health yaks. The xylitol produced by *Kurtzmaniella* is beneficial for curing and preventing various diseases, which has been approved for clinical application [73]. Overall, we found that microorganisms associated with threatening health increased in the gut of hens in the highest density. According to previous analyses, the potential reasons that includes (1) caged layers that might suffer in health, reflecting the susceptibility to pathologies such as infections, fractures, etc. [74], (2) excessive-high stocking density might increase the sensitivity to environmental stress and may change the intestinal microbiome [44].

In this longitudinal characterization of the gut microbiome, including bacterial and fungal communities, we have detected significant differences in the intestinal microbiome between young and old laying hens (AH vs. BH and AL vs. BL), with different abundances in various classifications of taxa. Compared with kids, the abundances of phyla Firmicutes (AH vs. BH), Elusimicrobiota (AL vs. BL), and Actinobacteria (AL vs. BL) in the intestinal microbiome of old hens significantly increased. In contrast, the proportion of Bacteroidota (AH vs. BH), Spirochaetota (AH vs. BH), and Euryarchaeota (AL vs. BL) decreased (*p* < 0.05 or *p* < 0.01). Both Firmicutes and Bacteroidetes are important phyla for the host because of the former’s contribution in degrading cellulose and fiber [75], while the latter’s important role in degrading proteins and carbohydrates [76]. It was reported that phylum Elusimicrobiota didn’t contain the essential genes required for encoding acetate [77], while Actinobacteriota was the one of the candidates for the evolutionary originated of sodN gene [58]. The proportion of phylum Euryarchaeota significantly drops with the age of the host, which was consistent with the current study [78]. Spirochaetota is significantly enriched in AH, which might be a disadvantageous signal because microorganisms belonging to this phylum threaten host health [79]. At the genus level, the percentage of several bacteria (*Treponema*, *Terrisporobacter*, *Faecalicoccus*, *Dialister*, *Pseudoramibacter*, *Solobacterium*, *Syntrophococcus*, *Lachnospiraceae_FCS020_group*, *Enterorhabdus*, *Methanosphaera*) and fungi (*Cylindrocarpon*, *Cutaneotrichosporon*, *Sporobolomyces*, and *Pseudogymnoascus*) in younger hens (AL vs. BL and AH vs. BH) were significantly increased compared with the older. In contrast, two bacteria (*Sellimonas* and *Mitsuokella*) and three fungi (*Eurotium*, *Wardomyces*, and *Cephalotheca*) had the opposite trend. However, among those species whose proportion decreased as the hens aged, most of them were reported as links to polymicrobial infection in clinical cases, such as *Clostridium’s* close relative, genus *Terrisporobacter* had been implicated in bloodstream infections [80]. However, the hens in the current gut microbiome survey were healthy regardless of age, suggesting that the intestinal microbiome’s composition and structure are likely unstable in the early stage. When considering the two driven factors (stocking density and age), it was  found that the gut of laying hens with the highest density (BL vs. BH) and the younger (AL vs. BL and AH vs. BH) was significantly enriched with one phylum (Bdellovibrionota) and two genera (*Eisenbergiella* and *Lachnoclostridium*), wherease *Oscillospiraceae_UCG-002* and *Erysipelotrichaceae_UCG-003* had the opposite trend. The increased proportion of inflammation-related bacterium (*Oscillospiraceae*) and *Erysipelotrichaceae_UCG-003* were linked to the sub-health status in the host [62, 81]. Xie et al. [71] documented that *Lachnoclostridium* had a crucial role in response to variations in intestinal proteins, while *Eisenbergiella* was reported to be connected with weight gain in humans [66]. These data conveyed the message that the colonization of the gut microbiome is a constantly changing and non-fixed ecosystem influenced by the factors such as stocking density and age. Our results agreed with the previous analysis [82].

## Conclusion

In sum, the excessive increased stocking density in Langya laying hens exacerbated adverse effects on egg quality and significantly decreased bodyweight, fallopian tube index, and egg weight, but had no adverse effects on the liver index. Except for the yolk colour score, the changes in measurements driven by age that had the opposite trend to stocking density. Similar to growth performance and egg quality differences, the shifts between housing densities are probably linked to environmental stress, anticipating higher pressure and mapping it onto the microbial structure and composition shifts. Overall, with increased stocking density, we observed a significant increase in taxa associated with threatening health. In contrast, the percentages of beneficial microorganisms involved in regulating intestine functions, reducing incidences of gut diseases and driving the evolution of the immune system, were significantly reduced. In addition, in this longitudinal characterization of the gut microbiome, including bacterial and fungal communities, we have detected significant differences in the intestinal microbiome between young and old laying hens. Among those species whose proportion decreased as the hens aged, most were linked with polymicrobial infection in clinical cases. Consequently, we speculated that excessive high density drove the abundance of bacteria and fungi connected with health problems. The gut microecology gradually reaches a mature and balance status with age.

## Data Availability

The original sequence data has been submitted to the Sequence Read Archive (SRA) (NCBI) with accession no. PRJNA759544.
